# Assessing the Testability of the Multi-Theory Model (MTM) in Predicting Vaping Quitting Behavior among Young Adults in the United States: A Cross-Sectional Survey

**DOI:** 10.3390/ijerph191912139

**Published:** 2022-09-25

**Authors:** Manoj Sharma, Kavita Batra, Ravi Batra, Chia-Liang Dai, Traci Hayes, Melinda J. Ickes, Tejinder Pal Singh

**Affiliations:** 1 Department of Social and Behavioral Health, School of Public Health, University of Nevada, Las Vegas, NV 89119, USA; 2 Department of Medical Education, Kirk Kerkorian School of Medicine, University of Nevada, Las Vegas, NV 89102, USA; 3 Office of Research, Kirk Kerkorian School of Medicine, University of Nevada, Las Vegas, NV 89102, USA; 4 Department of Information Technology, Coforge Ltd., Atlanta, GA 30338, USA; 5 Department of Teaching and Learning, College of Education, University of Nevada, Las Vegas, NV 89154, USA; 6 College of Nursing, School of Health Professions, University of Southern Mississippi, Hattiesburg, MS 39406, USA; 7 Department of Kinesiology and Health Promotion, College of Education, University of Kentucky, Lexington, KY 40509, USA; 8 Department of Family and Preventive Medicine, Division of Public Health, School of Medicine, University of Utah, Salt Lake City, UT 84108, USA

**Keywords:** vaping, smoking cessation, tobacco use, college students, young adults, e-cigarettes, nicotine, cannabis

## Abstract

Purpose: Given the increased exposure to e-cigarettes and nicotine among young adults, difficulty in quitting vaping is likely, which supports the need for effective behavioral interventions. Therefore, this cross-sectional study aims to assess the testability of the contemporary multi-theory model of health behavior change in predicting the vaping quitting behavior among young adults in the United States. Methods: A nationally representative sample of 619 young adults engaged in vaping behavior and aged 18–24 years was recruited to complete a 49-item web-based survey. A structural equation model was used to test relationships between MTM constructs. Hierarchical multiple regression was utilized to predict the variance in the initiation and sustenance of vaping quitting behavior by predictor variables, such as demographic characteristics, history of behaviors, and MTM constructs. Results: Of 619 respondents, over 75% were White and nearly 70% had educational attainment equal to high school or some college. In total, 62% of respondents were using nicotine, followed by 33.3% were using cannabis. About 80% of the respondents reported being engaged in drinking alcohol, and nearly 45% were engaged in cigarette smoking. The predictive effect of all MTM constructs on vaping quitting initiation (adjusted R^2^ = 0.417, F (23, 595) = 20.215, *p* < 0.001) and sustenance (adjusted R^2^ = 0.366, F (23, 595) = 16.533, *p* < 0.001) was statistically significant. Conclusions: The findings of this study point to the usability and applicability of MTM in operationalizing and developing vaping quitting behavior interventions targeting young adults.

## 1. Introduction

Vaping uses electronic devices that heat liquids or aerosolize a variety of products, such as nicotine, cannabis, glycerol, and flavoring, which are inhaled by users [1,2]. The popularity of vaping, also known as the use of electronic cigarettes and electronic nicotine delivery systems (ENDS), is rising [3]. In 2018, over 41 million individuals reported being engaged in vaping worldwide [4,5]. According to the 2018 estimates given by the Centers for Disease Control and Prevention (CDC), nearly 26% of young adults aged 18–24 years are e-cigarette users [6]. Regrettably, young adults comprise the greatest proportion of e-cigarette users [5,7]. According to the Substance Abuse and Mental Health Services Administration (SAMSHA), college students have the highest tobacco usage rates, with a prevalence of 20% or more among this population [8,9]. The 2017 Monitoring the Future survey among youth found that 7.4% vaped nicotine, 3.6% vaped marijuana, and 8% vaped for flavor [10]. The American College Health Association (ACHA) 2021 data highlight college students’ continued uptake, with 14.3% of undergraduate students reporting vaping over the last three months from the day of the survey [11].

Numerous studies performed at college campuses reported the increasing rates of e-cigarette use and nicotine dependence [8,12,13,14,15]. E-cigarettes (or electronic nicotine delivery systems) are much like traditional cigarettes in that they can create a dependency [16]. The nicotine dependency manifests in physical and mood-related symptoms including anxiety, irritability, and insomnia. More harmful problems from e-cigarette use have been reported. For instance, increased lung problems, such as chronic cough, wheezing, and other respiratory issues have been found to be associated with vaping [17]. Additionally, cognitive problems from e-cigarette use have manifested in the brains of users in their mid-twenties, including memory and concentration loss and brain fog [1,17,18]. Civeiletto and Hutchinson (2022) posited that the neurologic dangers from the nicotine in e-cigarettes are greater for teens and young adults, because their brain is still developing [1]. Xie and colleagues (2020) found that individuals engaged in vaping had a significantly higher cognitive dissonance than individuals who never smoked [18]. The popular brand, JUUL, is reported to have as much nicotine as twenty traditional cigarettes [1,19]. Such high levels of nicotine exposure are likely to lead to dependence and difficulty quitting [13]. Research on e-cigarette cessation, especially among young adults and college students, is minimal, given the recent rise in e-cigarette popularity [4,7,20,21].

Most of the studies on vaping quitting have been qualitative. A study by Antin and colleagues (2019) in a qualitative assessment found that youth perceive vaping as a means for reducing smoking-related harm and a tool for quitting smoking [22]. Sanchez and colleagues (2021) recounted evidence that suggests more than 40% of young adults and adolescents seeking to quit vaping tried within the past five years [4]. Their qualitative study reported several obstacles to quitting vaping such as easy availability, enjoying flavors, ability to be discreet, lack of awareness, lack of trustworthy information, and perception of social acceptance. Similarly, Hester and colleagues (2021) reported over half of those reporting current vaping intended to quit [20]. While it is encouraging that some young adults have intentions to quit, further understanding of factors to best support their quitting journey can be an important subject of investigation, especially when there are negative cues to action available in the form of new flavors and designs. In another qualitative study conducted by Amato and colleagues (2021), it was reported that health, money, relief from addiction, and betterment of social life were the prime motivators for quitting vaping among youth [23].

Vaping is appealing to adolescents and young adults because of the variety of flavors available, accessibility, and design [24]. Abadi and colleagues (2017) found that college students at a public university viewed the use of e-cigarettes, marijuana, and hookahs as less harmful than traditional cigarette smoking [12]. Youth who had never used e-cigarettes recognized greater physical and social risks associated with vaping. The authors identified that there had been limited interventions from health professionals and suggested counseling of college students may hinder the uptake and sustenance of e-cigarettes. To the best of our knowledge, only one other study has used a theoretical framework of the theory of planned behavior in a quantitative paradigm to explore quitting vaping behavior among young adults [20]. While the study reinforced the significance of perceived behavioral control, the authors also noted limitations in the measures, as they were not specific to the behavior of quitting.

**Figure 1:**
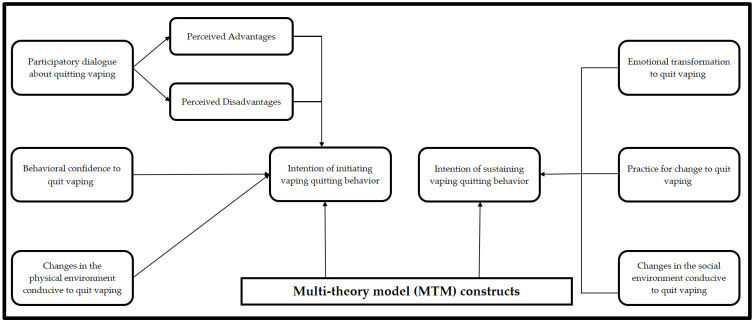
MTM framework used to measure vaping quitting behavior.

Therefore, this research seeks to investigate the testability of the contemporary Multi-theory Model (MTM) of health behavior change in predicting the initiation and sustenance of vaping quitting behavior. Additionally, the authors hypothesize that all MTM subscales (“participatory dialogue”, “behavioral confidence”, “changes in the physical environment”, “emotional transformation”, “practice for change”, and “changes in the social environment”) will have independent predictive influence on initiating and sustaining vaping quitting behavior after controlling demographic and other behavioral factors. The MTM is a fourth-generation theory and has been applied to predict the initiation and sustenance of various health behaviors among diverse groups. The theory has assessed factors affecting the behaviors of young adults including college students. Based on the results obtained from several cross-sectional studies [25,26,27,28,29,30,31,32,33] and health behavior interventions [34,35], applying the MTM to behaviors such as binge drinking, physical activity, stress management, meditative practices, and vaccine acceptance, the MTM seems efficacious in determining the likelihood of starting and maintaining a myriad of health behaviors. The MTM framework (Figure 1) has two main constructs of “initiation” and “sustenance”. The construct of “initiation” further has three subscales of “participatory dialogue”, “behavioral confidence”, “changes in the physical environment” [34,35]. Similarly, the construct of sustenance (i.e., long-term behavior changes) has three subscales, namely “emotional transformation”, “practice for change”, and “changes in the social environment”.

## 2. Materials and Methods

### 2.1. Study Design and Participants

This analytical cross-sectional study was conducted in April–May 2022 to recruit individuals who met the following criteria: aged 18–24 years, residing in the United States (U.S), those engaged in vaping behavior, and those who had the ability to understand English. Efforts were made to recruit a nationally representative sample by demographic variables, such as gender, race, ethnicity, and region.

### 2.2. Data Collection and Sampling

Data for this study were collected from 25 April–4 May 2022 using a web-based survey built into an online survey software; Qualtrics (Provo, UT, USA. https://www.qualtrics.com, accessed on 1 April 1 2022). Data collection for this study was conducted by the Qualtrics research marketing team [36,37]. The survey link was distributed among the audience panel members of Qualtrics through various methods, including listserv, in-app notifications, and through specialized campaigns to recruit “hard to reach” population groups. The Qualtrics research team includes experienced fielding experts to ensure fast and accurate data collection. First, Qualtrics collected an initial 10–20 completed responses for study investigators to review. This first wave of results came from real respondents and was termed a “soft launch”. This allowed us to understand how the survey questions are being interpreted by survey respondents [36,37]. It also ensures the accuracy of survey flow and logic as intended. Once approved by the study investigators, a “full launch” of the data collection was established to attain the final sample size (N = 619) with a census balance as indicated earlier to ensure the representativeness of the sample [36,37,38]. Data collection efforts were continued until the quotas of the study were met. To mirror the census representation (Census ref), the following quota constraints were applied (Table 1). 

### 2.3. Ethical Considerations

This study was approved as exempt research by the Institutional Ethics Committee of the University of Nevada, Las Vegas (protocol ID: UNLV-2022-142) on 15 March 2022. All data collected in this study were deidentified and the privacy of the participants was ensured. All study participants received a comprehensive explanation of the study aims, objectives, data collection procedure, risks, and benefits. Every participant was given informed consent through which they could agree or disagree to participate in this study. Participation was completely voluntary and participants were allowed to leave the study at any time. Strategies, such as the use of unique digital fingerprinting and “prevent ballot box stuffing” were used by the Qualtrics team to prevent duplicated responses. Participants were asked some screening questions (without disclosing the specific details of the study and inclusion criteria) at the beginning to determine their eligibility to prevent any self-selection bias. Incentives in the form of gift cards, redeemable points, cash rewards, vouchers, or SkyMiles were given to the participants, who completed the survey. These incentives were given per the contract between Qualtrics and panel providers.

### 2.4. Survey Instrument

The survey tool used in this study was grounded in the MTM framework [39,40]. The MTM tool has two main components, namely “initiation”, and “sustenance”. The initiation component has three constructs, “participatory dialogue”, “behavioral confidence”, and “change of physical environment”, which seek to explain the commencement of the behavior (Figure 1). Participatory dialogue relies on the “perceived advantages” of starting the behavior outweighing the “perceived disadvantages” of declining to start the behavior [39,40]. The construct of “behavioral confidence” requires that one’s own belief to implement the behavior increases and “changes in the physical environment” suggest that likelihood of behavior initiation is influenced by proper surroundings and space. Sustenance, on the other hand, has 3 constructs too, namely “emotional transformation”, “practice for change”, and “changes in the social environment”, to maintain the behavior [39,40]. Emotional transformation occurs when the individual transcends the negative “chatter” occurring in the psyche and practice for change involves monitoring actions towards goal attainment and adjusting when deviating from the purpose. The final construct, changes in the social environment, suggests that support from important persons (i.e., spouse, friends, and trusted health providers) can sustain the behavior. The work continues to evaluate the ability of the MTM to explain the initiation and sustenance of vaping cessation behaviors. Identifying the factors that may move young adults towards vaping cessation behaviors can inform much-needed interventions that reduce the negative impact of using electronic cigarettes and support cessation efforts.

A 49-item survey was developed to measure the above-mentioned constructs related to vaping quitting behavior. The survey included 18 items related to the demographic and behavioral characteristics, five items to measure “perceived advantages”, five items to measure “perceived disadvantages”, five items for the “behavioral confidence”, five items for the “change in the physical environment”, three items for the “emotional transformation”, three items to measure “practice for change”, three items for the “change in the social environment”, and two items to measure initiation and sustenance. Except for the items of “perceived advantages”, “perceived disadvantages”, “initiation”, and “sustenance”, which were measured on the 5-point likelihood scale (ranging from ‘not at all likely’ to ‘completely likely’), all other items were measured on the surety scale (ranging from ‘not at all sure’ to ‘completely sure’ on 5-point Likert scale).

### 2.5. Face and Content Validity of the Survey Instrument

**Figure 2:**
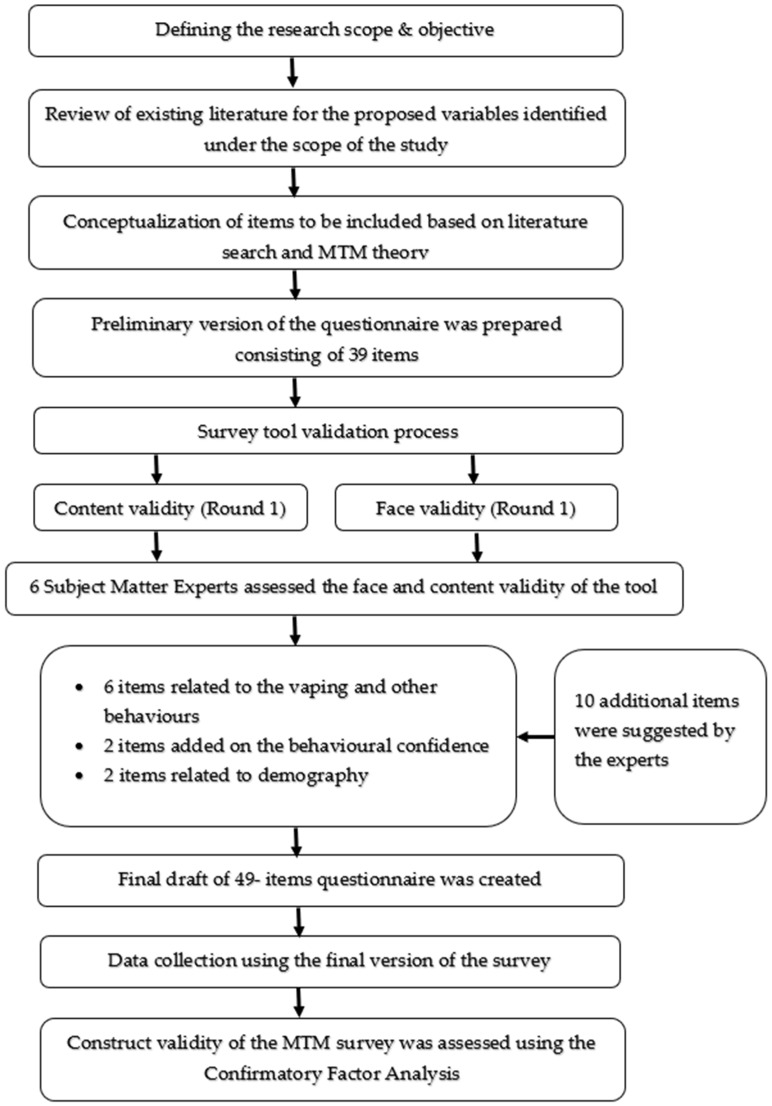
A flow chart depicting the designing and validation of the questionnaire.

After the development of the initial version of the survey, the face and content validity of the survey was assessed by six subject matter experts in the fields of behavioral theories, substance-abuse research, and survey validation. The initial version included a total of 39 items. Experts provided the comments to improve the readability/clarity of the survey and 10 additional items were suggested to include in the survey tool. Of these 10 items, 6 items were related to vaping or other concomitant behaviors. Two items were added to the construct of “behavioral confidence” and the remaining 2 items were added to the demographic questions (Figure 2). The feedback of experts was effectively addressed in two rounds before finalizing the survey instrument.

### 2.6. Sample Justification

For the sample calculation, the following formula: n = (z)2 *p* (1 − *p*)/d2 was used where z is the value from the standard normal distribution reflecting the confidence level that was used (e.g., Z = 1.96 for 95%), d is the margin of error and *p* is the population proportion. Inputs, such as a 95% confidence interval (alpha = 0.05, z score = 1.96), d = 5%, the proportion of vaping among young adults aged 18–24 years was 25.8% per data reported by the National Center for Health Statistics, Centers for Disease Control and Prevention in 2018 [CDC] [6], and the total estimated population of 18–24 years adults (303,960,02) were used to calculate the sample [38]. The minimum sample required was 325 (295 + 10% non-response), and the sample size used in this study was reasonably larger to investigate hypothesized effects and for conducting structural equation modeling [41].

### 2.7. Data Analysis

A confirmatory factor analysis was performed to assess the construct validity of the survey. A structural equation model generated through AMOS was used to test relationships. A good fitting model is accepted if the value of the CMIN/df is <5, the Tucker and Lewis index (TLI); the Confirmatory fit index (CFI) is >0.90 [42,43]. In addition, an adequate fitting model was accepted if the AMOS computed value of the root mean square error approximation (RMSEA) lower bound and upper bound values are under 0.05 and 0.08, respectively [43].

**Figure 3:**
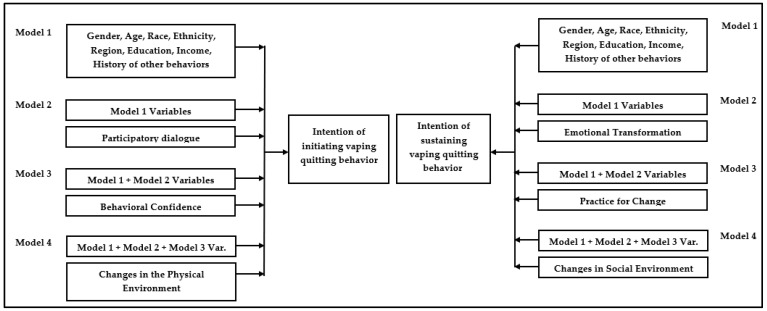
A flowchart detailing the model building process of the hierarchical regression.

The responses of the survey were analyzed using univariate, bivariate as well as multivariate statistical methods. Categorical variables were represented as counts and proportions, whereas continuous variables (if normally distributed) were represented as means and standard deviations. In the univariate analysis, the 95% confidence intervals of proportions were computed through the binomial “exact” method. The intercorrelation matrix among continuous variables was ascertained using Pearson’s bivariate correlation test. Two separate models of the hierarchical multiple regression were built to predict or explain the variance in the dependent variables (e.g., initiation and sustenance) by independent variables, such as demographic characteristics, history of behaviors, and MTM constructs. A complete model-building process is described in Figure 3 as shown below. All assumptions of regression, including linearity, independence of residuals, homoscedasticity, multicollinearity, and normality were assessed.

## 3. Results

### 3.1. Structural Model Assessment 

#### 3.1.1. Initiation

**Figure 4:**
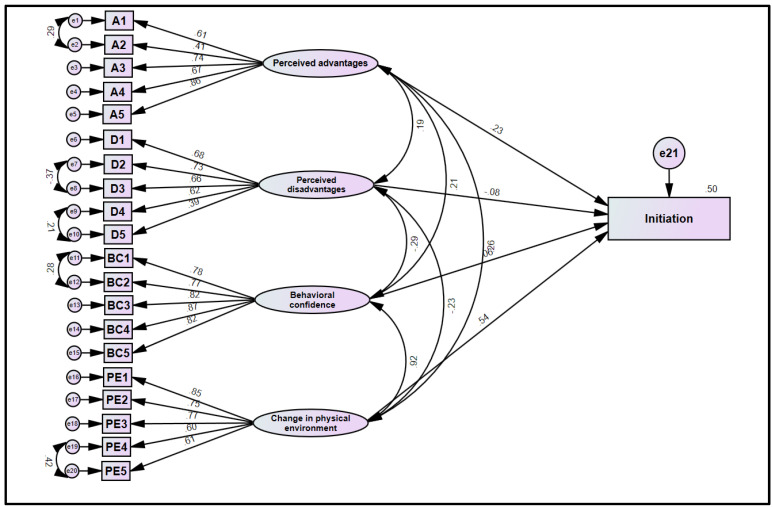
Structural model of initiating vaping quitting behavior.

The fit indices for the initiation model were in the range of acceptability: CMIN/df = 4.1, TLI = 0.91, CFI = 0.92, and RMSEA = 0.07 (0.061–0.071). The squared multiple correlation (R^2^) was 0.50 for initiation, which shows that 50% variance in the initiation is accounted by “perceived advantages”, “perceived disadvantages”, “behavioral confidence”, and “changes in the physical environment”. The study assessed the relationships of “perceived advantages”, “perceived advantages”, “behavioral confidence”, and “changes in the physical environment” on initiation. The relationship of “perceived advantages” on initiation was positive and significant (β = 0.23, t = 5.912, *p* < 0.001), which supports the hypothesized model. The relationship of “perceived disadvantages” on initiation was negative and marginally significant (β = −08, t = −2.035, *p* = 0.04). The relationship of “behavioral confidence” on initiation was positive but insignificant (β = 0.060, t = 0.449, *p* = 0.6). The relationship of “changes in the physical environment” on initiation was positive and significant (β = 0.540, t = 3.963, *p* < 0.001), which supports the hypothesized model (Figure 4).

Figure description: The standardized loadings in this model are correlations between the indicator or manifest variables (in boxes) and the latent factors (ovals). The double-headed arrows among the latent factors (ovals) represent correlations. The double-headed arrows between error terms represent correlations among the errors. A1-A5 represents 5 items of “perceived advantages”, D1-D5 represents 5 items of “perceived advantages”, BC1-BC5 represents 5 items of “Behavioral confidence”, and PE1-PE5 represents the 5 items of “Changes in the Physical Environment”.

#### 3.1.2. Sustenance

**Figure 5:**
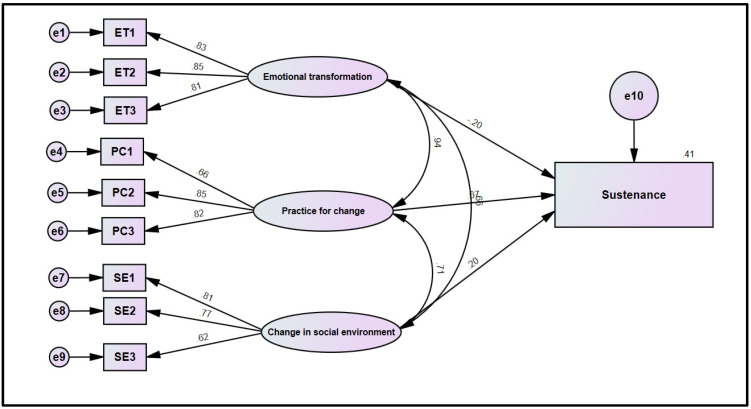
Structural model for sustaining vaping quitting behavior.

For the sustenance model, all the fit indices were consistent with the conventional thresholds for an acceptable fitting model. The fit indices for the sustenance model were: CMIN/df = 3.1, TLI = 0.97, CFI = 0.98, and RMSEA = 0.05 (0.045–0.072). The squared multiple correlation (R^2^) was 0.41 for sustenance, which shows that 41% variance in the sustenance is accounted by “emotional transformation”, “practice for change”, and “change in the social environment”. This model assessed the relationships of “practice for change”, “emotional transformation”, and “changes in the social environment” on sustenance. The relationship of “emotional transformation” on sustenance was negative and insignificant (β = −0.20, t = −1.037, *p* = 0.3), which does not support the hypothesis. The relationship of “practice for change” on sustenance was positive and significant (β = 0.670, t = 3.184, *p* = 0.001). The relationship of “changes in the social environment” on sustenance was positive and significant (β = 0.196, t = 3.169, *p* = 0.002), which supports the hypothesized model (Figure 5).

Figure description: The standardized loadings in this model are correlations between the indicator or manifest variables (in boxes) and the latent factors (ovals). The double-headed arrows among the latent factors (ovals) represent correlations. The double-headed arrows between error terms represent correlations among the errors. ET1-ET3, PC1-PC3, and SE1-SE3 represent 3 items each of the “Emotional transformation”, “Practice for Change”, and “Changes in the Social Environment”, respectively.

### 3.2. Reliability Diagnostics

The results of reliability diagnostics indicated that the Cronbach alpha values for each MTM construct: “perceived advantages”, “perceived disadvantages”, “behavioral confidence”, “changes in the physical environment”, “emotional transformation”, “practice for change”, and “changes in the social environment” were 0.81, 0.75, 0.91, 0.86, 0.87, 0.81, and 0.78, respectively. The reliability of the entire scale was 0.91. Further analysis indicated that the Mcdonald’s omega (ω) values for each MTM construct: “perceived advantages”, “perceived disadvantages”, “behavioral confidence”, “changes in the physical environment”, “emotional transformation”, “practice for change”, and “changes in the social environment” were 0.82, 0.76, 0.91, 0.86, 0.87, 0.81, and 0.78, respectively. The coefficient omega of the entire scale was 0.91. ${\left(\sum^{\;}\lambda_{i}\right)}^{2}\;$= 355.6242; where λ is an indicator for the factor loadings and $\sum^{\;}V\left(e_{i}\right)\;$= 32.833; where *V* is a residual error variance.

$$\omega =\frac{{\left(\sum^{\;}\lambda_{i}\right)}^{2}}{{\left(\sum^{\;}\lambda_{i}\right)}^{2}+\sum^{\;}V\left(e_{i}\right)\;}=\frac{355.6242}{355.6242+32.833}=0.915$$

### 3.3. Demographic and Behavioral Characteristics

The sample mirrored the census representation by gender, race, ethnicity, and region. Over 75% of the sample were White. The mean age of the sample was 21.74 ± 1.6 years. Around 70% of respondents had educational attainment equal to high school or some college with no degree (Table 2). Nearly 65% of respondents were employed and the majority of the sample had annual income under $100,000.

As indicated in Table 3, 62% of our sample were nicotine users, followed by 33.3% of cannabis users. About 80% of respondents reported being engaged in drinking alcohol and about 45% were engaged in cigarette smoking. Over 90% of the sample reported having at least one friend who vaped, and over 50% of respondents reported vaping behavior among their family members.

### 3.4. Mean Values of MTM Constructs

All mean scores with possible and observed ranges of all MTM constructs are shown in Table 4. These statistics indicate the mean scores for all constructs for sustenance were relatively lower than the constructs of initiation, which highlight the need of developing behavioral interventions to bring a long-term change.

### 3.5. Intercorrelation Matrix

The results of Pearson correlation test indicate a direct and moderately strong correlation of “behavioral confidence” with “changes in the physical environment”, “emotional transformation”, and “practice for change” (*p* < 0.001, Table 5). The “changes in the physical environment” was strongly and directly correlated with the “emotional transformation” and “practice for change”. All intercorrelations are shown in Table 5.

### 3.6. Hierarchical Multiple Regression

When controlled for demographic and behavioral characteristics, the addition of MTM constructs in the following models led to a statistically significant increase in the predictability of the vaping quitting behavior. In the initiation model (Table 6), model 4 explained 41.7% of the variance in the vaping quitting behavior. The full model of demographic, behavioral, and MTM variables to predict vaping quitting initiation behavior was statistically significant, R^2^ = 0.439, F (23, 595) = 20.215, *p* < 0.001; adjusted R^2^ = 0.417. The addition of behavioral confidence to the prediction of initiating vaping quitting behavior (Model 3) led to a statistically significant increase in R^2^ of 0.279, F (1, 596) = 280.31, *p* < 0.001. The addition of participatory dialogue to the prediction of initiating vaping quitting behavior (Model 2) also led to a statistically significant increase in R^2^ of 0.08, F (1, 597) = 52.455, *p* < 0.001. Demographic and behavioral factors were not significant. With each unit increase in the participatory dialogue, behavioral confidence, and changes in the physical environment, the initiation of vaping quitting behavior increased by 0.03, 0.08, and 0.07 units, respectively.

For the sustenance, model 4 explained 36.6% of variance in the vaping quitting behavior, R^2^ = 0.390, F (23, 595) = 16.533, *p* < 0.001; adjusted R^2^ = 0.366 (Table 7). The addition of practice for change to the prediction of sustenance (Model 3) led to a statistically significant increase in R^2^ of 0.066, F (1, 596) = 62.013, *p* < 0.001. The addition of emotional transformation to the prediction of sustenance (Model 2) also led to a statistically significant increase in R^2^ of 0.253, F (1, 597) = 215.25, *p* < 0.001. With each unit’s increase in the emotional transformation, practice for change, and changes in the social environment, the sustenance of vaping quitting behavior increased by 0.05, 0.131, and 0.08 units, respectively. Demographic and behavioral factors were insignificant in the sustenance model.

## 4. Discussion

The purpose of this study was to identify theoretically driven correlates of vaping quitting behavior among young adults in a sample of the US population. Among participants in our sample who were engaged in vaping behavior, the majority (62%) were using nicotine while a third were using cannabis. A study based on 2017 Monitoring the Future Survey [10] in a sample of 14,560 youth found that 8.0% were vaping just for flavor, while 7.4% vaped nicotine and 3.6% vaped marijuana. Therefore, of the total 19% of young adult vapers in that study, 39% were vaping nicotine and 19% were vaping cannabis. According to the CDC estimates in 2018, nearly 26% of young adults aged 18–24 years reported being engaged in vaping [6]. While our study did not aim to gather the prevalence of vaping, the findings could imply a continued trend of increased risk for both nicotine and cannabis vaping among youth, warranting intervention efforts to support both prevention and quitting.

Among the correlates that explained the intent of initiating vaping quitting behavior, it was found that all three constructs of MTM were significant predictors and explained a substantial proportion of the variance (42%), according to the normative standards in social and behavioral sciences [28]. Behavioral confidence emerged as the largest contributor to the model. This construct has been found to be a strong predictor in many MTM-based studies with different behaviors [25,26,30,32,34,44]. It is important to build this construct by helping young adults who already vape start quitting in small steps that may entail (1) setting a future quit date; (2) breaking down the process of quitting into manageable stages; (3) exploring all possible sources that would build the confidence for quitting such as self-sureness, from powerful others, from Almighty, from a deity as appropriate in some cultural groups, etc.; and (4) building the confidence to identify and overcome potential impediments in quitting vaping. These approaches have been suggested for smoking cessation [45], but their extension to vaping cessation will be unique.

The construct of participatory dialogue is also crucial in motivating and keeping the motivation levels high for quitting vaping and was supported by this study. This construct has been found to be a strong predictor in many MTM studies [26,32]. In interventions, this may require emphasizing benefits to health, saving money, improving image, especially in front of peers and others, not becoming dependent, and enjoying life more. At the same time it would be important to downplay some potential disadvantages of quitting such as (1) the relaxation that they may be deriving (here suggesting alternative relaxation methods can be helpful); (2) the socialization that they may be enjoying (here alternative ways of socializing can be underscored); (3) the craving associated with quitting (here emphasizing the temporary nature of the craving); (4) losing friends (here emphasizing the nature of true and well-wishing friends). These advantages and disadvantages are in line with previous findings from qualitative studies as identified by Sanchez et al. (2021) and Amato et al. (2021). However, additional research is needed to determine what would best support quitting behaviors among young adults as integrated into future interventions [3,23].

The third initiation construct in MTM is changes in the physical environment which has been corroborated as a significant construct with several behaviors and found to be statistically significant in this study [25,30,32,34,35,46]. For influencing changes in the physical environment related to quitting vaping, getting rid of all vaping devices from the environment, not buying any vaping devices, substituting vaping time with something else, avoiding social media that triggers vaping behavior, and increasing the ability to overcome marketing pressures at the individual level can be helpful. Additionally, for large-scale interventions, comprehensive policy support may be an important factor to consider.

Among the correlates that explained the intent of sustaining or maintaining quitting vaping behavior, all three constructs of MTM were found to be statistically significant predictors and explained a substantial proportion of variance (37%), according to the normative standards in social and behavioral sciences [28]. The emotional transformation was the largest contributor to the model. This construct has also been found to be a significant predictor in many MTM studies with different behaviors [25,30,32,44,45]. For modifying emotional transformation, it is important to direct feelings toward the goal of quitting vaping, developing self-motivation, and overcoming self-doubt. Practice for change was also found to be significant in this study like many other studies [25,30,31]. This construct can be fostered through monitoring of quitting behavior which can be done through keeping a diary, a log, use of an app, or other such means. It also requires one to reflect on the barriers one is encountering and adapt coping strategies accordingly. The construct of changes in the social environment was found to be significant in this study and has also been found to be significant in many other studies [30,31]. This construct can be modified by announcing the quitting plans to family and friends and mobilizing their support. Likewise, support from health professionals, social media, etc. can also be utilized in this regard.

In terms of descriptive data, the study found that about 45% of the young vapers also smoked cigarettes and 82% drank alcohol. While no national data are available that delineate the usage of these three behaviors together. The Youth Risk Behavior Surveillance System collects data separately for these behaviors [47]. However, these findings are in consonance with vaping becoming a “gateway drug” for youth [48]. Despite the limitations of sample selection in our study, overall, these are alarming statistics and behoove both practitioners and researchers to focus on decreasing the experimentation, polysubstance use, and subsequent dependence on these two gateway drugs among young adults that have deleterious effects on lifespan and healthy living. The study found that 92% of the young vapers had a close friend who was a vaper. This has important programmatic implications for designing peer-to-peer interventions and creating anti-vaping peer pressure which at present seems to be flowing in the wrong direction. Further, 36% reported suffering from adverse mental health outcomes and 43% reported suffering from negative physical health outcomes. These data should be sufficient for policymakers to institute firm tobacco control measures which include all vaping products to protect youth from vaping. However, there are practical difficulties in this regard. A study by Pignataro and Daramola (2020) reported that college students replaced tobacco with vaping, using electronic cigarettes, and using hookahs [8]. The study revealed that the students had a less favorable position when discussing a ban on electronic cigarettes and smokeless tobacco than conventional tobacco products. Students in this study felt that ‘vaping was not technically tobacco or smoke’ and therefore wanted it to be excluded from the tobacco policy. There is a need to correct misperceptions of vaping harm and to determine how that impacts attitudes toward policy implementation (and compliance).

### 4.1. Implications for Practice

There is a need for tailored interventions to support quitting vaping among young adults. Further understanding of the best setting and approach is needed. However, it is clear that coordinated efforts are critical, which may include collaborations with wellness centers of colleges/universities, healthcare facilities, community-based organizations including recreation centers, faith-based organizations, worksites, and other such places which reach and can support young adults. Based on the findings of this study, there is a need to incorporate methods to build behavioral confidence, participatory dialogue, changes in the physical environment, emotional transformation, practice for change, and changes in the social environment. For influencing participatory dialogue, the emphasis on key advantages and downplaying of disadvantages; for building behavioral confidence the delineation of steps; for fostering changes in the physical environment the getting rid of vaping devices; for emotional transformation directing feelings toward quitting; for practice for change, monitoring vaping quitting behavior; and for changes in the social environment fostering social support as key strategies have been discussed earlier. Future research is needed to help determine the most appropriate learning methods (e.g., small and large group discussions, role plays, simulations, and psychodrama) and approach (e.g., one-on-one counseling, group classes, text-based services).

Given the significance of the physical environment in this study, there is a need to support environmental changes related to quitting vaping. While a focus on individual-level behaviors (e.g., encouraging individuals to get rid of or not buy devices) is important, health promotion efforts can also support policy and organizational changes. These areas warrant further investigation to determine the potential impact on both vaping initiation and continued use among young adults.

### 4.2. Strengths and Limitations of the Study

Our study is among the few studies that have utilized a contemporary theoretical framework in a quantitative paradigm to identify the determinants of vaping quitting behavior. Hence, the findings of this study can be used to design vaping quitting interventions. However, our study had some limitations. Our sample, though nationally representative for gender, national region, race, and ethnicity, was not representative of other dimensions, which could have influenced the results. This attributes to limited generalizability. Next, we only sampled individuals who vape and could not compare the characteristics with those who do not vape. Further, we did not differentiate those who vaped just for flavor, as our study focused on nicotine and cannabis. Future studies can be planned to investigate demographic differences among groups who vape vs. who do not. Our study used a cross-sectional design and hence we cannot make firm conclusions due to a lack of the ability to establish temporal associations. Finally, self-reported data have several limitations. Future research must undertake interventional work using randomized controlled designs.

## 5. Conclusions

This study, the first of its kind, identified the antecedents of quitting vaping behavior utilizing the multi-theory model (MTM) of health behavior change. All three constructs of MTM in the initiation model and all three constructs of MTM in the sustenance model were significant predictors and were responsible for substantial explanatory power for quitting vaping among youth. The MTM is ready for the operationalization and development of vaping quitting behavior interventions among youth. Such educational interventions should be developed and tested to determine their efficacy in a variety of settings in which young adults can be reached.

## Figures and Tables

**Table 1:** Census distribution by gender, race, ethnicity, and region.

Demographic Characteristic	Census Distribution, Population Parameters (%)
Gender	Male: 48%; Female: 52%; Non-binary: natural fallout
Race	White (~75%); Black/AA (~13%); Asian or Pacific Islander (~6%); American Indian/Alaskan Native/Other (~6%)
Ethnicity	Hispanic (~18%); Non-Hispanic (~82%)
Region	Northeast: 17%; Midwest: 21%; West: 24%; South: 38%

**Table 2:** Socio-demographics characteristics of the sample engaged in vaping (N = 619).

Variable	Categories	Descriptive Statistic	95% CI (LCL, UCL)
Gender	Female	322 (52.0)	48.0, 56.0
	Male	289 (46.7)	42.7, 50.7
	Other	8 (1.3)	0.6, 2.5
Age in years (M ± SD)	-	21.74 ± 1.6	21.61, 21.87
Race	American Indian or Alaska Native	10 (1.7)	0.8, 3.0
	Asian	36 (6.0)	4.2, 8.1
	Black or African American	81 (13.4)	10.8, 16.4
	White	463 (76.5)	72.9, 79.9
	Others including the multiethnic origin	15 (2.5)	1.4, 4.1
Ethnicity	Hispanic	111 (17.9)	15.0, 21.2
	Non–Hispanic	508 (82.1)	78.8, 85.0
Region	Midwest	132 (21.3)	18.2, 24.8
	Northeast	107 (17.3)	14.4, 20.5
	South	238 (38.4)	34.6, 42.4
	West	142 (22.9)	19.7, 26.5
Education	College Degree (Associate or Bachelors)	133 (21.4)	18.3, 24.9
	Graduate Degree	27 (4.4)	2.9, 6.3
	High school graduate (or equivalent including GED)	229 (37.0)	33.2, 40.9
	Some college but no degree	201 (32.5)	28.8, 36.3
	Other	29 (4.7)	3.2, 6.7
Employed	Yes	402 (64.9)	61.0, 68.7
	No	217 (35.1)	31.3, 39.0
* Hours worked (Per week) (M ± SD)	-	32.41 ± 11.5	31.18, 33.64
Income	Less than $ 50,000	315 (50.9)	46.9, 54.9
	$ 50,000 to $ 100,000	225 (36.3)	32.6, 40.3
	$100,001 to $150,000	52 (8.4)	6.3, 10.9
	$150,001 to $200,000	18 (2.9)	1.7, 4.6
	More than $200,000	9 (1.5)	0.7, 2.7

Note. M = Mean; SD = Standard deviation; CI = Confidence interval; LCL = Lower confidence limit; UCL = Upper confidence limit; * Hours worked/week calculated for those being employed.

**Table 3:** Summary of variables related to vaping and other behaviors’ history (N = 619).

Variable	Categories	Frequencies (Percentages)	95% CI (LCL, UCL)
Vape Type	Cannabis	206 (33.3)	29.6, 37.1
	Nicotine	384 (62.0)	58.1, 65.9
	Other	29 (4.7)	3.2, 6.7
Do you smoke cigarettes	Yes	276 (44.6)	40.6, 48.6
	No	343 (55.4)	51.4, 59.4
Do you drink alcohol	Yes	510 (82.4)	79.2, 85.2
	No	109 (17.6)	14.8, 20.8
How many of your closest friends vape	One or more	570 (92.1)	89.7, 94.1
	None	49 (7.9)	5.9, 10.3
Family members who vape	Yes	326 (52.7)	48.6, 56.7
	No	293 (47.3)	43.3, 51.4
Suffered from any mental health outcome as a result of vaping	Yes	221 (35.8)	32.0, 39.7
	No	397 (64.2)	60.3, 68.0
Suffered from any physical health outcome as a result of vaping	Yes	265 (42.8)	38.9, 46.8
	No	354 (57.2)	53.2, 61.1

Note. CI = Confidence interval; LCL = Lower confidence limit; UCL = Upper confidence limit.

**Table 4:** Mean score and ranges of MTM constructs.

Variables	Possible range (Min, Max)	Range (Min, Max)	Mean ± SD	Skewness	Kurtosis
Intent of Initiation	(0, 4)	(0, 4)	1.89 ± 1.32	0.112	−1.058
1. Perceived advantages	(0, 20)	(0, 20)	11.43 ± 4.83	−0.089	−0.702
2. Perceived disadvantages	(0, 20)	(0, 20)	7.50 ± 4.52	0.239	−0.556
3. Behavioral Confidence	(0, 20)	(0, 20)	8.84 ± 6.02	0.176	−0.835
4. Changes in the Physical Environment	(0, 20)	(0, 20)	10.09 ± 5.56	0.045	−0.668
Intent of Sustenance	(0, 4)	(0, 4)	1.74 ± 1.35	0.184	−1.086
5. Emotional Transformation	(0, 12)	(0, 12)	5.98 ± 3.55	0.043	−0.726
6. Practice for Change	(0, 12)	(0, 12)	5.77 ± 3.43	0.088	−0.715
7. Changes in the Social Environment	(0, 12)	(0, 12)	6.05 ± 3.40	−0.046	−0.637

**Table 5:** Pearson correlations between MTM constructs used in this study.

Variables	1	2	3	4	5	6
1. Participatory Dialogue	1	0.25 **	0.29 **	0.32 **	0.27 **	0.25 **
2. Behavioral Confidence	0.25 **	1	0.77 **	0.74 **	0.72 **	0.50 **
3. Changes in the Physical Environment	0.29 **	0.77 **	1	0.80 **	0.76 **	0.59 **
4. Emotional Transformation	0.32 **	0.74 **	0.80 **	1	0.79 **	0.55 **
5. Practice for Change	0.27 **	0.72 **	0.76 **	0.79 **	1	0.60 **
6. Changes in the Social Environment	0.25 **	0.50 **	0.59 **	0.55 **	0.60 **	1

** *p* < 0.01; “Participatory dialogue” was measured through “perceived advantages” and “perceived disadvantages”.

**Table 6:** Hierarchical multiple regression to predict the likelihood of initiation of vaping quitting (N = 619).

Variables	Model 1		Model 2		Model 3		Model 4	
	B	β	B	β	B	β	B	β
Constant	3.241 **	-	2.542 *	-	1.275	-	0.888	-
Age	−0.023	−0.028	−0.019	−0.024	−0.017	−0.020	−0.012	−0.014
Gender: Male (Ref: Female)	0.096	0.036	0.071	0.027	0.055	0.021	0.013	0.005
Other gender (Ref: Female)	−0.996 *	−0.085	−0.740	−0.063	−0.695	−0.059	−0.630	−0.054
Race: Non-White (Ref: White)	0.103	0.034	0.143	0.047	−0.018	−0.006	0.008	0.003
Ethnicity: Non-Hispanic (Ref: Hispanic)	−0.280	−0.081	−0.141	−0.041	−0.062	−0.018	−0.042	−0.012
Region: Northeast (Ref: Midwest)	0.059	0.017	0.062	0.018	0.051	0.015	0.052	0.015
South	0.177	0.065	0.134	0.049	0.165	0.061	0.196	0.072
West	0.060	0.019	0.071	0.023	0.145	0.046	0.156	0.049
Education (Ref: Associate or Bachelors)								
Graduate Degree	0.166	0.026	0.177	0.027	0.078	0.012	0.133	0.021
High school graduate or equivalent	−0.127	−0.046	−0.044	−0.016	−0.019	−0.007	−0.057	−0.021
Some college but no degree	−0.164	−0.058	−0.099	−0.035	−0.129	−0.046	−0.202	−0.072
Other	0.085	0.014	0.209	0.033	0.041	0.007	−0.014	−0.002
Income: $ 50,000 to $ 100,000 (Ref: <$50,000)	−0.017	−0.006	−0.022	−0.008	0.003	0.001	−0.017	−0.006
$100,001 to $150,000	0.102	0.021	0.129	0.027	−0.036	−0.007	−0.023	−0.005
$150,001 to $200,000	0.060	0.008	0.128	0.016	−0.262	−0.033	−0.347	−0.044
More than $200,000	−0.020	−0.002	−0.168	−0.015	−0.399	−0.036	−0.392	−0.035
Cigarette smoking (Ref: No)	−0.099	−0.037	−0.020	−0.008	0.088	0.033	0.087	0.033
Alcohol consumption (Ref: No)	−0.20	−0.058	−0.182	−0.052	−0.247 *	−0.071	−0.214	−0.062
Vaping among friends (Ref: No)	−0.417 *	−0.085	−0.30	−0.061	−0.097	−0.020	−0.089	−0.018
Vaping among family (Ref: No)	−0.195	−0.074	−0.083	−0.032	−0.013	−0.005	0.018	0.007
Participatory dialogue	-	-	0.064 **	0.290	0.036 **	0.163	0.03 **	0.136
Behavioral confidence	-	-	-	-	0.123 **	0.560	0.076 **	0.345
Changes in the physical environment	-	-	-	-	-	-	0.069 **	0.291
R^2^	0.050	-	0.127	-	0.406	-	0.439	-
F	1.568	-	4.120 **	-	18.514 **	-	20.215 **	-
Δ R^2^	0.050	-	0.077	-	0.279	-	0.033	-
Δ F	1.568	-	52.455 **	-	280.310 **	-	34.646 **	-

* *p*-value < 0.05; ** *p*-value < 0.001; Adjusted R^2^ of Model 4 = 0.417.

**Table 7:** Hierarchical multiple regression to predict the likelihood for the sustenance of vaping quitting (N = 619).

Variables	Model 1		Model 2		Model 3		Model 4	
	B	β	B	β	B	β	B	β
Constant	2.113 *	-	0.878	-	0.339	-	0.029	-
Age	4	0.011	0.007	0.008	0.013	0.015	0.018	0.022
Gender: Male (Ref: Female)	0.12	0.045	0.103	0.038	0.095	0.035	0.1	0.037
Other gender (Ref: Female)	−0.516	−0.043	−0.121	−0.01	−0.083	−0.007	−0.236	−0.02
Race: Non-White (Ref: White)	0.112	0.036	0.017	0.005	−0.043	−0.014	−0.03	−0.01
Ethnicity: Non-Hispanic (Ref: Hispanic)	−0.331 *	−0.094	−0.228	−0.065	−0.197	−0.056	−0.163	−0.046
Region: Northeast (Ref: Midwest)	0.175	0.049	0.179	0.05	0.22	0.062	0.182	0.051
South	0.273	0.098	0.229	0.083	0.237	0.085	0.225	0.081
West	0.225	0.07	0.229	0.071	0.261	0.081	0.228	0.071
Education (Ref: Associate or Bachelor’s)								
Graduate Degree	0.135	0.02	−0.085	−0.013	−0.006	−0.001	0.063	0.009
High school graduate or equivalent	−0.155	−0.056	−0.224	−0.08	−0.149	−0.053	−0.116	−0.042
Some college but no degree	−0.331 *	−0.115	−0.376 *	−0.131	−0.269 *	−0.094	−0.29 *	−0.101
Other	−0.069	−0.011	−0.181	−0.028	−0.091	−0.014	−0.085	−0.013
Income: $ 50,000 to $ 100,000 (Ref: <$50,000)	0.031	0.011	0.033	0.012	0.015	0.005	0.011	0.004
$100,001 to $150,000	0.039	0.008	−0.034	−0.007	−0.055	−0.011	−0.02	−0.004
$150,001 to $200,000	−0.112	−0.014	−0.345	−0.043	−0.443	−0.055	−0.44	−0.055
More than $200,000	−0.065	−0.006	−0.245	−0.022	−0.126	−0.011	−0.212	−0.019
Cigarette smoking (Ref: No)	−0.163	−0.06	−0.091	−0.034	−0.116	−0.043	−0.1	−0.037
Alcohol consumption (Ref: No)	0.001	0.003	0.02	0.006	0.073	0.021	0.056	0.016
Vaping among friends (Ref: No)	−0.309	−0.062	−0.266	−0.053	−0.187	−0.037	−0.218	−0.044
Vaping among family (Ref: No)	−0.114	−0.042	0.017	0.006	0.026	0.01	0.048	0.018
Emotional transformation	-	-	0.195 **	0.512	0.069 *	0.181	0.054 *	0.141
Practice for change	-	-		-	0.167 **	0.425	0.131 **	0.332
Changes in the social environment	-	-		-		-	0.082 **	0.206
R^2^	0.045	-	0.298	-	0.364	-	0.390	-
F	1.416	-	12.082 **	-	15.531 **	-	16.533 **	-
Δ R^2^	0.045	-	0.253	-	0.066	-	0.026	-
Δ F	1.416	-	215.257 **	-	62.013 **	-	24.889 **	-

* *p*-value < 0.05; ** *p*-value < 0.001; Adjusted R^2^ for Model 4 = 0.366.

## Data Availability

The data presented in this study are available on request from the corresponding author. The data are not publicly available due to ethical reasons.
